# Orange Juice and Yogurt Carrying Probiotic *Bacillus coagulans* GBI-30 6086: Impact of Intake on Wistar Male Rats Health Parameters and Gut Bacterial Diversity

**DOI:** 10.3389/fmicb.2021.623951

**Published:** 2021-04-01

**Authors:** Carine N. Almada-Érix, Caroline N. Almada, Lucélia Cabral, Viviane Priscila Barros de Medeiros, Aline R. Roquetto, Valfredo A. Santos-Junior, Melline Fontes, Any Elisa S. S. Gonçalves, Andrey dos Santos, Pablo C. Lollo, Marciane Magnani, Anderson S. Sant’Ana

**Affiliations:** ^1^Department of Food Science and Nutrition, Faculty of Food Engineering, University of Campinas, Campinas, Brazil; ^2^Institute of Biosciences, Department of General and Applied Biology, São Paulo State University (UNESP), Rio Claro, Brazil; ^3^Laboratory of Microbial Processes in Food, Department of Food Engineering, Technology Center, Federal University of Paraíba, João Pessoa, Brazil; ^4^Department of Physical Education, Federal University of Great Dourados, Dourados, Brazil; ^5^Research Informatics Core, Research Resource Center, University of Illinois at Chicago, Chicago, IL, United States; ^6^Microbial Resources Division, Research Center for Chemistry, Biology and Agriculture (CPQBA), University of Campinas, Campinas, Brazil; ^7^Internal Medicine Department, Faculty of Medical Sciences, University of Campinas, Campinas, Brazil

**Keywords:** spore-forming bacteria, beneficial microbes, intestinal microbiome, functional food, fermented food

## Abstract

This study aimed to investigate the impact of the food matrix (orange juice and yogurt) on the effects of the spore-forming probiotic microorganism *Bacillus coagulans* GBI-30 6086 in health parameters and gastrointestinal tract (gut) bacterial diversity in *Wistar* male rats. Rats (*n* = 48) were randomly distributed into six groups. The groups were the Control (which received sterile distilled water), Juice (which received orange juice), Yogurt (which received yogurt), Probiotic *Bacillus* (which received *B. coagulans* GBI-30 6086 in distilled water), Probiotic Juice (which received orange juice with *B. coagulans* GBI-30 6086), and Probiotic Yogurt (which received yogurt with *B. coagulans* GBI-30 6086). Each animal belonging to the different groups was treated for 21 days. The daily administration of probiotic juice or probiotic yogurt did not affect the rats’ food or body weight. Rats fed with Probiotic Yogurt showed lower glucose and triglycerides levels (*p* < 0.05) in comparison to the control group (*p* < 0.05), while no changes in these parameters were observed in the rats fed with Probiotic Juice. Rats fed with Probiotic Yogurt showed a higher gut bacterial diversity than the control group (*p* < 0.05), and higher abundance (*p* < 0.05) of *Vibrionales*, *Enterobacteriales*, *Burkholderiales*, *Erysipelotrichales*, and *Bifidobacteriales* compared to all other groups. No changes were observed in the expression levels of antioxidant enzymes or heat shock protein 70 of rats fed with probiotic yogurt or probiotic juice. Results reveal that the consumption of yogurt containing *B. coagulans* GBI-30 6086 decreases triglycerides and glucose levels and positively impacts the gut bacterial ecology in healthy rats. These animal model findings indicate that the matrix also impacts the functionality of foods carrying spore-forming probiotics. Besides, this research indicates that yogurt is also a suitable food carrier of *Bacillus coagulans* GBI-30 6086.

## Introduction

Probiotics are defined as live microorganisms that, when administered in adequate amounts, confer health benefits on the host (Hill et al., 2014). *Bifidobacterium, Lactobacillus*, and amended genera have been the main probiotic microorganisms incorporated in food matrices. However, there is a growing interest in probiotic *Bacillus coagulans* and *B. subtilis* in foods (Cutting, 2011; Soares et al., 2019). Probiotic *Bacillus* (PB) is resistant to several unit operations used during food processing. It survives better under adverse gastric and intestinal conditions than non-spore-forming probiotics due to the spores’ greater resistance (Fouad et al., 2017; Cao et al., 2020).

Health benefits related to the consumption of PB include prevention and treatment of gastrointestinal diseases (Dolin, 2009), modulation of the intestinal microbiota (Sun et al., 2011), immune modulation, and relief of lactose intolerance symptoms (Kimmel et al., 2010). Studies have reported intestinal microbiota modulation by PB in cereals-mix fermented (Ng et al., 2013) and positive effects of PB incorporated in milk on immune response (Sun et al., 2011).

For the successful application in food matrices, probiotics must survive the processing and also during storage. The food matrix must transport and deliver the cells to the gastrointestinal tract (gut; Fazilah et al., 2018). The interaction of probiotics with food components is directly linked to the carrier matrix’s physicochemical and nutritional characteristics (Soares et al., 2019). Specific components of the food matrix may confer protection during the storage and when cells are exposed to several stresses such as low pH, bile acids, and digestive enzymes (Fazilah et al., 2018).

Dairy products are food matrices widely explored for the incorporation of probiotics. Even though yogurt is considered a suitable matrix for the delivery of probiotic bacteria (Rutella et al., 2016), the probiotic yogurts’ stability is related to technological operations the probiotic bacteria are subjected and their intrinsic resistance to stresses (Granato et al., 2010). The oxidative stress induced by the formation of reactive oxygen species such as superoxide ion or hydrogen peroxide affects the viability of *Bifidobacterium*, *Lactobacillus*, and amended genera in yogurts. Thus, the probiotic yogurt’s shelf life is limited by post-acidification during storage, which causes a loss of viability of probiotic cells due to the persistent metabolic activity of starter lactic acid bacteria (Xu et al., 2015). The addition of glucose oxidase to yogurt during processing and the use of packages with low oxygen permeability rates have been proposed as alternatives to control these tasks (Cruz et al., 2013), but they increase the costs of the final product. Therefore, the development of probiotic yogurts carrying spore-forming probiotic strains appears a promising strategy.

On the other hand, fruit juices are perceived as healthy and refreshing beverages well-accepted by consumers of all ages. These beverages have been suggested as matrices for incorporating probiotics because they are interesting for vegans and consumers interested in low cholesterol foods (Pereira and Rodrigues, 2019). However, some factors can limit the probiotics’ viability in fruit juices, such as low pH, oxygen, presence of multiple antimicrobial components, and treatment systems used in processing (Pimentel et al., 2019). Otherwise, the survival of probiotics in fruit juices may be enhanced by the absence of prior fermentation (and interaction with starter cultures), relatively fast passage through the gastrointestinal, and naturally occurring juice constituents (e.g., fibers, sugars, vitamins, minerals, and phenolics; Filho et al., 2019).

Previous studies reported the survival of PB during food processing, storage, and or exposure to *in vitro* digestion when incorporated in different non-dairy matrices such as tea (Majeed et al., 2019), jelly candies (Miranda et al., 2020), dried date pastes (Marcial-Coba et al., 2019), and orange juice (Soares et al., 2019). However, no prior studies have explored PB’s functionality incorporated in fruit juice and the impacts of the food matrix on the health benefits of spore-forming probiotics *in vivo*.

Therefore, the present study was performed to assess the effects of *Bacillus coagulans* GBI-30 6086, a spore-forming bacterium presenting GRAS status and claimed probiotic properties (FDA, 2016, 2017) on biochemical parameters and gut microbiota ecology of healthy rats when incorporated in yogurt and orange juice.

## Materials and Methods

### Probiotic Strain

The probiotic strain *B. coagulans* GBI-30, 6086 was kindly donated by the Ganeden Biotech Inc., (Mayfield Heights, Ohio, United States) as a powder containing the spores. It is a safe strain (Endres et al., 2009; Salvetti et al., 2016) available on the market with recognized benefits to humans (Cao et al., 2020) with a potential for application in a range of foods (Almada-Érix et al., 2021). The whole-genome shotgun project was deposited in the DDBJ/EMBL/GenBank under the accession number (JPSK00000000; Orrù et al., 2014).

### Food Matrix Preparation and Inoculation of *B. coagulans* GBI-30 6086

Orange juices were prepared using commercially concentrated pulp. The total soluble solids content was adjusted to 11°Brix with water, following pasteurization at 95°C for 30 s in a water bath Quimis, model 0334M-28 (Diadema, SP, Brazil). *B. coagulans* GBI-30, 6086 spores were inoculated after the thermal processing.

The yogurt production was performed according to the procedures described by Tamime and Robinsons (2007). Milk standardized to total solids (13%) was subjected to thermal treatment (90°C/5 min) in a water bath Quimis, model 0334M-28 (Diadema, SP, Brazil) and cooled down to 42°C. Then, traditional lactic culture (*Streptococcus thermophilus* and *Lactobacillus bulgaricus*, CHR-Hansen, Brazil) was added at 2.5% (v/v), the following fermentation in a kiln (Marconi, model MA 032, Piracicaba, SP, Brazil) at 45°C until pH reached 4.6 and cooled to 10°C. For the preparation of the probiotic yogurt, *Bacillus coagulans* GBI-30, 6086 spores were added after fermentation. For the Probiotic *Bacillus* group, *Bacillus coagulans* GBI-30, 6086 spores were added to sterile distilled water. In all groups, the final concentration was 10^8^ spores/ml. Probiotic *Bacillus*, probiotic juice, and probiotic yogurt were prepared weekly and stored at 4°C.

The probiotic bacteria strain presented counts around 10^8^ CFU/ml in juice and yogurt throughout the 21 days of refrigerated storage (data not shown). The enumeration of *Bacillus coagulans* GBI-30, 6086 in yogurt or juice comprised the application of a heat shock at 80°C/10 min, followed by pour plate in Glucose Yeast Extract Agar (BC) and incubation at 40°C/48 h under aerobiosis. Further details on the formulation of Glucose Yeast Extract Agar are available in Soares et al. (2019).

### Chemical Composition of Food Matrices

After preparation, the samples were submitted to moisture, and ash contents were determined according to standard methods described by the AOAC (2012) and total lipids following the Institute Adolfo Lutz (IAL, 2005) methods in all food matrices. Total proteins were determined in juices according to the Association of Official Analytical Chemists (AOAC, 2012) and in yogurts following the IAL (2005) method. The total carbohydrates content was estimated by difference.

### Experimental Design Using Wistar Rats

The Ethical Commission previously approved all experimental procedures on Animal Use (CEUA, UNICAMP, São Paulo, Brazil, protocol n° 3456-1). A total of 48 male Wistar rats at 21 days (specific pathogen-free) were obtained from the Animal Breeding Center (University of Campinas, UNICAMP, SP, Brazil), and were used in the study. The animals were kept in individual cages under a specific condition (22 ± 1°C, 12 h photoperiod; 60–70% relative humidity) with food (AIN 93 M diet, Nutivital, São Paulo) and water provided *ad libitum* (Reeves et al., 1993) for adaptation during 3 weeks. The animals were randomly distributed into six groups of eight animals as follows: (a) Control group, which received sterile distilled water; (b) Juice, which received orange juice; (c) Yogurt, which received yogurt; (d) Probiotic *Bacillus*, which received *B. coagulans* GBI-30 6086 suspended in distilled water; (e) Probiotic Juice, which received orange juice with *B. coagulans* GBI-30 6086; and (f) Probiotic Yogurt, which received yogurt with *B. coagulans* GBI-30 6086.

All animal groups received a volume of four milliliters of liquid daily administered by orogastric gavage during 21 days. The administred volume was defined considering the volume of 1 ml/100 g according to the official protocols (Andersen et al., 2004).

Weight gain was monitored weekly, and the food intake was assessed every 2 days. After the 21-days of the experiment, six milliliters of blood were collected *via* direct cardiac puncture from anesthetized with an intraperitoneal injection of 1 ml of ketamine hydrochloride (75 mg) and 1 ml of xylazine hydrochloride (5 mg) per kg of body weight. Samples were centrifuged at 3,000 × *g*, 10 min, 4°C. Animals were euthanized, and the gastrocnemius muscle and cecum fecal samples were carefully removed and stored at −80°C (Costa et al., 2019).

### Assessment of Biochemical Parameters in Wistar Rats

Blood samples (4 ml) were collected *via* direct cardiac puncture and centrifuged (3,000 × *g*, 10 min, 4°C) from anesthetized animals. Serum biochemical parameters were determined using commercial kits according to the manufacturer’s instructions (Labcenter®, Tocantins, Brazil): aspartate aminotransferase (AST), alanine aminotransferase (ALT), total cholesterol, high-density lipoprotein (HDL), triglycerides, uric acid, creatinine, glucose, total protein, and albumin.

### Western Blot Analyses

Analyses of expression of heat shock protein 70 (HSP70) and endogenous antioxidant enzymes, namely superoxide dismutase (SOD), catalase (CAT), and glutathione peroxidase (GPx), were performed according to Moura et al. (2016), with minor adaptations. Gastrocnemius muscle sample (200 mg) of each animal in each experimental group was homogenized in five volumes of extracting buffer (200 mmol/L EDTA, 1 mol/L Tris-Base, 10 mmol/L orthovanadate, 2 mmol/L phenylmethanesulfonyl fluoride, 10 mmol/L sodium pyrophosphate, 0.1 mg/ml aprotinin, 100 mmol/L sodium fluoride, Triton 10%, ultrapure water) using Polytron (Pro Scientific Model Pro 200). The mixture was centrifuged at 14,000 × g for 40 min at 4°C, and the supernatant was collected. The extracts were subjected to SDS-PAGE (8%) and transferred using a semi-dry system (Bio-Rad, CA, United States) to a nitrocellulose membrane of 0.22 μm. The nitrocellulose membranes were blocked with 3% bovine serum albumin (BSA) and incubated with specific primary antibodies overnight.

To assess the protein level, the appropriate secondary antibodies were used for detection. [HSP 70 (#ADI-SPA-810, Enzo life sciences - Farmingdale, United States), SOD (#AB51254, Abcam, Cambridge United Kingdom), GPx (#AB22604, Abcam, Cambridge UK), CAT (#AB1877, Abcam, Cambridge United Kingdom)]. A molecular weight standard was used and run concurrently on each gel to determine the antibody’s proper molecular weight. Immunoreactive bands were detected by chemiluminescence (Super Signal West Pico Chemiluminescent Substrate Kit, Thermo Scientific, United States). The bands were visualized using the UNITEC instrument (model Alliance LD2), and blots were quantified using the UN SCAN IT software (Moura et al., 2016). Results were expressed as % in comparison to the control value.

### Gut Microbiota Ecology Analysis

Total DNA was extracted from the cecal contents with the QIAmp DNA Stool Kit. For profiling microbiome composition, the hyper-variable region (V3–V4) of the bacterial 16S rRNA gene was amplified using the Illumina 16S Metagenomic Sequencing Library Preparation guide Illumina 16S metagenomic sequencing library preparation (Illumina Technical Note 15044223), which uses the following sequence: 338F - 5'- TCGTCGGCAGCGTCAGATGTGTATAAGAGACAGCCTA CGGGNGGCWGCAG -3 and 785R - 5'- GTCTCGTGGGCTCGGAGA TGTGTATAAGAGACAGGACTACHVGGGTATCTAATCC -3'. Using 300 bp paired readings and MiSeq v3 reagents, each law’s ends were overlaid to generate high-quality complete readings of the V3 and V4 regions. More than 100,000 readings per sample were generated, commonly recognized as sufficient for metagenomic research. The sequencing was performed in the Illumina Miseq equipment (Neoprospecta Consulting and Research SA, Santa Catarina state, Brazil).

### Taxonomic Assignment Obtained by 16S rRNA Gene Sequencing Analysis

Initial sequences quality check was performed by the FASTQC and then quality filtering using the Trimmomatic (0.36; Bolger et al., 2014). The search for chimera was performed using the UCHIME2 (Edgar et al., 2011). The sequences were then analyzed using the QIIME (quantitative insights into microbial ecology), version 1.9.0 software (Caporaso et al., 2010). OTUs were clustered at 97% identity using the available reference approaches (UCLUST algorithm; Edgar, 2010) and identity against the Green genes bacterial 16S rRNA database (13_5 release; McDonald et al., 2012) using the RDPII classifier (Wang et al., 2007) and PyNast for aligning sequences (Caporaso et al., 2010).

For annotation analysis, all OTUs observed less than two times (i.e., singletons) were discarded. The samples’ rarefaction was performed (normalization for the same number of OTUs—45,035 OTUs). The rarefied data were used for alpha diversity evaluation through the QIIME to generate rarefaction curves, Good’s coverage, Chao1 richness (Chao and Bunge, 2002), and Shannon and Simpson diversity indices (Shannon, 1948; Simpson, 1949). Beta diversity was evaluated with the UniFrac (Lozupone and Knight, 2005). Feature and sample clustering were simultaneously analyzed using the heat map exploratory data analysis tool in the XLSTAT software version 2015.2 (Adinsoft, Paris, France). Annotated sequences were deposited and are available at the National Center for Biotechnology Information (NCBI^1^; BioProject PRJNA631217).

### Statistical Analysis

Data of centesimal composition are presented as mean ± standard deviation (SD). Results were analyzed by ANOVA, followed by the Scott-Knott test considering *p* < 0.05, using the Sisvar software 5.6 (Lavras, MG, Brazil). All other data were presented as means and the standard error of the mean (SEM) and analyzed by ANOVA, followed by the Duncan *post-hoc* test considering a p < 0.05 using the statistical package for social sciences (SPSS, Chicago, IL, United States) software, version 23.0 for windows.

## Results

### Chemical Composition of Food Matrices

The moisture, ashes, total lipids, proteins, and total carbohydrates did not differ (*p* ≥ 0.05) between the juice and probiotic juice or between the yogurt and probiotic yogurt (Supplementary Table 1). However, contents of ashes, lipids, proteins, and estimated total carbohydrates differ (*p* < 0.05) between the yogurt and juice and between the probiotic yogurt and probiotic juice. Yogurt and probiotic yogurt showed total ashes, lipids, and proteins contents higher than the juice and probiotic juice (*p* < 0.05). Otherwise, the total estimated carbohydrates in the juice and probiotic juice were higher (*p* < 0.05) than the yogurt and probiotic yogurt.

### Diet Intake, Body Weight, and Biochemical Parameters

The daily administration of juice or yogurt probiotic did not affect the food intake (568.9 g ± 23.3 and 537.6 ± 20) or the bodyweight of rats (334.0 g ± 9.0 and 330.2 ± 7.7), respectively, in comparison to the control groups (*p* ≥ 0.05; Table 1).

**Table 1:** Bodyweight and food intake of healthy *Wistar* male rats after 21 days of administration of juice, probiotic juice, yogurt, and probiotic yogurt.

Parameter (g)	Group^*^					
	Control	Juice	Yogurt	Probiotic *Bacillus*	Probiotic juice	Probiotic yogurt
Bodyweight	335.9 ± 8.0^a^	331.7 ± 6.2^a^	341.1 ± 6.9^a^	329.6 ± 7.8^a^	334.0 ± 9.0^a^	330.2 ± 7.7^a^
Food intake	593.6 ± 20.2^a^	597.6 ± 13.2^a^	551.4 ± 18.2^a^	582.2 ± 24.6^a^	568.9 ± 23.3^a^	537.6 ± 20.5^a^

* Groups were as follows: Control: received distilled water; Juice: received orange juice; Yogurt: received yogurt; probiotic *Bacillus*: received *B. coagulans* GBI-30 6086 suspended in distilled water; Probiotic Juice: received orange juice with *B. coagulans* GBI-30 6086; and Probiotic Yogurt: received yogurt with *B. coagulans* GBI-30 6086. Data are expressed as means ± SEM. Different superscript letters on the same line indicate statistical differences by the Duncan test (*p* < 0.05).

The consumption of probiotic yogurt for 21 days reduced classical health parameters in rats, such as glucose (9.82%) and triglycerides (34.66%) serum levels in comparison to the control group (*p* < 0.05). The probiotic *Bacillus* group (which received the probiotic in distilled water) also showed a significant reduction in triglycerides (23.85%) serum levels when compared to the control group (*p* < 0.05). It was interesting to note that glucose and triglycerides did not change in the rats fed with probiotic juice (Table 2). The other measured parameters (ALT, AST, creatinine, uric acid, cholesterol, HDL, albumin, and total protein) did not change (*p* ≥ 0.05) in the probiotic groups (rats that received probiotic *Bacillus* in distilled water, probiotic yogurt, or probiotic juice) when compared to the control groups (rats that received yogurt, juice, or distilled water; Table 2).

**Table 2:** Effect of probiotic yogurt and probiotic juice consumption during 21 days on healthy *Wistar* male rats’ biochemical parameters.

Parameter	Group^*^					
	Control	Juice	Yogurt	Probiotic *Bacillus*	Probiotic juice	Probiotic yogurt
Glucose (mg/dL)	133.4 ± 4.8^a^	131.8 ± 2.8^ab^	131.5 ± 2.8^ab^	125.8 ± 3.4^ab^	126.9 ± 6.4^ab^	120.3 ± 1.6^b^
Triglycerides (mg/dL)	85.1 ± 10.3^a^	70.4 ± 5.2^ab^	63.2 ± 10.7^ab^	48.6 ± 5.4^b^	64.8 ± 11.3^ab^	55.6 ± 4.7^b^
Cholesterol (mg/dL)	54.2 ± 3.1^a^	50.5 ± 3.7^a^	58.3 ± 2.2^a^	48.6 ± 3.7^a^	51.2 ± 2.5^a^	48.6 ± 3.5^a^
HDL (mg/dL)	40.8 ± 3.5^a^	40.8 ± 4.4^a^	40.0 ± 3.9^a^	40.8 ± 4.0^a^	40.2 ± 3.1^a^	40.2 ± 3.9^a^
Total protein (g/dL)	5.9 ± 0.32^a^	5.7 ± 0.17^a^	5.5 ± 0.16^a^	5.5 ± 0.14^a^	5.5 ± 0.15^a^	5.6 ± 0.13^a^
Albumin (g/dL)	3.7 ± 0.31^a^	3.7 ± 0.33^a^	4.0 ± 0.45^a^	3.9 ± 0.15^a^	4.1 ± 0.43^a^	4.1 ± 0.34^a^
ALT (U/L)	9.3 ± 1.0^ab^	7.8 ± 0.56^a^	8.8 ± 0.48^ab^	11.5 ± 1.1^b^	9.9 ± 0.92^ab^	10.6 ± 2.1^ab^
AST (U/L)	28.0 ± 2.7^a^	28.9 ± 1.2^a^	35.5 ± 2.7^a^	34.0 ± 2.6^a^	37.0 ± 3.9^a^	36.4 ± 4.2^a^
Creatinine (mg/dL)	0.33 ± 0.02^ab^	0.30 ± 0.03^a^	0.37 ± 0.02^ab^	0.37 ± 0.04^ab^	0.38 ± 0.02^ab^	0.42 ± 0.03^b^
Uric acid (mg/dL)	0.81 ± 0.09^ab^	0.82 ± 0.07^ab^	0.77 ± 0.07^a^	1.0 ± 0.13^ab^	1.1 ± 0.14^b^	0.99 ± 0.11^ab^

* Groups were as follows: Control: received distilled water; Juice: received orange juice; Yogurt: received yogurt; probiotic *Bacillus*: received *B. coagulans* GBI-30 6086 suspended in distilled water; Probiotic Juice: received orange juice with *B. coagulans* GBI-30 6086; and Probiotic Yogurt: received yogurt with *B. coagulans* GBI-30 6086. Data are expressed as means ± SEM. Different superscript letters on the same line indicate statistical differences by the Duncan test (*p* < 0.05).

### Protein Expression

The consumption of probiotic juice, probiotic yogurt, or *Bacillus* in distilled water did not change the expression of antioxidant enzymes (SOD, GPx, CAT) or HSP 70 in rats when compared to the control group (*p* < 0.05; Supplementary Figure 1).

### 16S rRNA Gene Sequencing-Based Structure of the Microbiota

A total of 21,466.032 reads were generated from the Next-Generation Sequencing (NGS) of amplicons corresponding to the V3–V4 region of the bacterial 16S rRNA gene. A total of 19.771.488 reads passed the sequence quality filters applied through the Trimmomatic (0.36) software, with an average value of 681.775 reads per sample after the quality filtering was obtained (Supplementary Table 2). The alpha-diversity and richness through the number of ace, Chao1, Good’s estimated sample coverage (ESC), OTUs, and Shannon and Simpson indices were obtained for all the samples (Table 3).

**Table 3:** Alpha-diversity metrics (ace, Chao1, Good’s estimated sample coverage (ESC), OTUs, Shannon and Simpson indices) obtained for fecal samples of healthy *Wistar* male rats after 21 days of administration of probiotic yogurt and probiotic juice inferred from the sequencing of 16S V3–V4 amplicons.^*^

Treatments group	ace	Chao1	ESC	OTUs	Shannon	Simpson
Control	15,204.3	14,245.5	0.94	3,905.8	7.77	0.98
Juice	15,966.8	15,141.7	0.94	4,069.0	7.96	0.99
Yogurt	15,144.3	14,089.4	0.94	3,895.6	7.74	0.98
Probiotic *Bacillus*	15,197.9	14,053.6	0.94	3,983.0	7.75	0.98
Probiotic juice	16,191.7	15,171.4	0.94	4,014.6	7.84	0.98
Probiotic yogurt	18,788.5	17,518.4	0.92	4,852.2	8.29	0.99

* Groups were as follows: Control: received distilled water; Juice: received orange juice; Yogurt: received yogurt; probiotic *Bacillus*: received *B. coagulans* GBI-30 6086 suspended in distilled water; Probiotic Juice: received orange juice with *B. coagulans* GBI-30 6086; and Probiotic Yogurt: received yogurt with *B. coagulans* GBI-30 6086.

**Figure 1:**
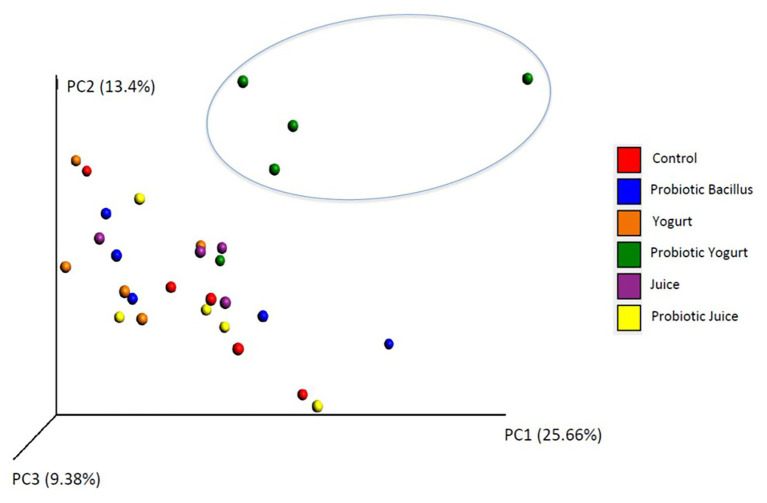
Principal coordinate analysis of jackknifed unweighted UniFrac distances for the 16S rRNA gene sequence data. Groups were as follows: Control: received distilled water; Juice: received orange juice; Yogurt: received yogurt; probiotic *Bacillus*: received *B. coagulans* GBI-30 6086 suspended in distilled water; Probiotic Juice: received orange juice with *B. coagulans* GBI-30 6086; and Probiotic Yogurt: received yogurt with *B. coagulans* GBI-30 6086.

In general, the Probiotic Yogurt group showed higher values for all alpha diversity indices than the other groups (Table 3). Notably, the highest values of Chao1 indices were found in the Probiotic Yogurt and Probiotic Juice groups (17518.4 and 15171.4, respectively). The six treatments’ alpha diversity data (Control, Juice, Yogurt, Probiotic *Bacillus*, Juice probiotic, and Yogurt probiotic) was analyzed using Kruskal-Wallis. There was a statistical difference in OTUs, Chao1, and Shannon indices among all the treatments (*p* < 0.05; Supplementary Table 3). The estimated sample coverage was satisfactory for 90% of the samples. The results of the analysis of the beta diversity, based on the unweighted Uni-Frac analysis, indicated that the Probiotic Yogurt samples formed a discrete group distinguished from the other five groups (Yogurt, Juice, Probiotic Juice, Probiotic *Bacillus*, and Control; Figure 1). When the Permanova statistical analysis was performed using the beta-diversity data, statistical differences were observed among the treatments (*p* < 0.01; Supplementary Table 4).

### Taxonomic Assignment Obtained by 16S rRNA Gene Sequencing Analysis

The taxonomic assignment obtained by 16S rRNA gene sequencing analysis showed that the OTUs belonged to three major different bacterial classes in the six groups treatments (Yogurt; Probiotic Yogurt; Juice; Probiotic Juice; Probiotic *Bacillus*; Control): *Clostridia* (from 51.8 to 46.4%), followed by *Bacteroidia* (from 43.3 to 33.8%), and *Bacilli* (from 10.1 to 5.5%) with no statistical differences (*p* ≥ 0.05). On the other hand, *Gammaproteobacteria* (4.6%) and *Betaproteobacteria* (1.1%) showed a high abundance (*p* < 0.05) in the Probiotic Yogurt treatment (Table 4).

**Table 4:** Relative abundance of bacterial classes inferred from 16S rRNA gene sequencing analysis.^*^

Taxonomy (%)	Control	Juice	Yogurt	Probiotic *Bacillus*	Probiotic juice	Probiotic yogurt
*Clostridia*	47.5 ± 9.5^a^	49.2 ± 3.7^a^	51.8 ± 5.0^a^	47.3 ± 8.7^a^	46.9 ± 6.5^a^	46.4 ± 7.5^a^
*Bacteroidia*	41.7 ± 8.9^a^	38.9 ± 3.7^a^	33.8 ± 5.0^a^	41.6 ± 7.2^a^	43.3 ± 6.2^a^	39.3 ± 4.7^a^
*Bacilli*	7.7 ± 3.5^a^	8.0 ± 2.2^a^	10.1 ± 2.1^a^	7.3 ± 2.2^a^	7.0 ± 1.0^a^	5.5 ± 2.5^a^
*Gammaproteobacteria*	0.4 ± 0.0^b^	0.5 ± 0.2^b^	0.3 ± 0.1^b^	0.4 ± 0.3^b^	0.3 ± 0.1^b^	4.6 ± 4.0^a^
*Betaproteobacteria*	0.4 ± 0.2^b^	0.7 ± 0.3^ab^	0.3 ± 0.2^b^	0.3 ± 0.2^b^	0.3 ± 0.1^b^	1.6 ± 1.1^a^
*Erysipelotrichi*	0.2 ± 0.1^a^	0.5 ± 0.2^a^	0.1 ± 0.0^a^	0.1 ± 0.1^a^	0.1 ± 0.1^a^	0.5 ± 0.4^a^
*Deltaproteobacteria*	0.2 ± 0.1^a^	0.3 ± 0.1^a^	0.3 ± 0.1^a^	0.4 ± 0.2^a^	0.3 ± 0.1^a^	0.3 ± 0.1^a^
*Mollicutes*	0.3 ± 0.3^a^	0.3 ± 0.2^a^	0.9 ± 1.1^a^	0.2 ± 0.1^a^	0.3 ± 0.2^a^	0.3 ± 0.2^a^
*Epsilonproteobacteria*	0.3 ± 0.2^a^	0.3 ± 0.2^a^	0.2 ± 0.1^a^	0.4 ± 0.4^a^	0.4 ± 0.4^a^	0.2 ± 0.1^a^
*Cyanobacteria;c_*	0.6 ± 0.3^a^	0.6 ± 0.7^a^	0.4 ± 0.5^a^	0.2 ± 0.1^a^	0.3 ± 0.2^a^	0.1 ± 0.1^a^

* Groups were as follows: Control: received distilled water; Juice: received orange juice; Yogurt: received yogurt; probiotic *Bacillus*: received *B. coagulans* GBI-30 6086 suspended in distilled water; Probiotic Juice: received orange juice with *B. coagulans* GBI-30 6086; and Probiotic Yogurt: received yogurt with *B. coagulans* GBI-30 6086; Data are expressed as means ± SEM. Different superscript letters on the same line indicate statistical differences by Tukey test (*p* < 0.05).

**Figure 2:**
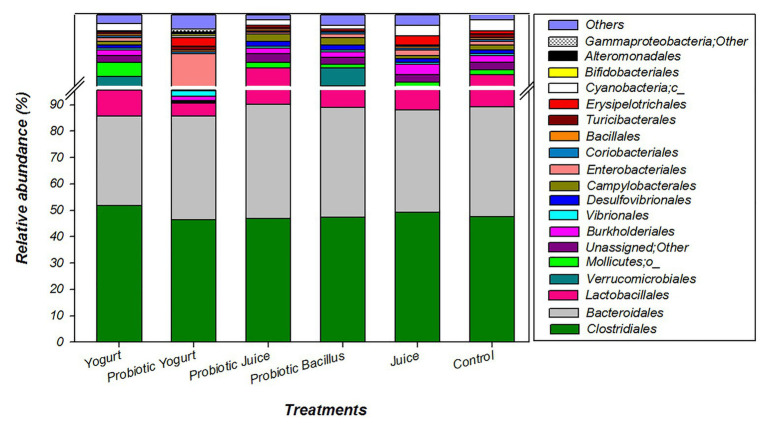
Relative abundance of bacterial groups inferred from 16S rRNA gene amplicon sequencing analysis from samples of each experimental or control group. Groups were as follows: Control: received distilled water; Juice: received orange juice; Yogurt: received yogurt; probiotic *Bacillus*: received *B. coagulans* GBI-30 6086 suspended in distilled water; Probiotic Juice: received orange juice with *B. coagulans* GBI-30 6086; and Probiotic Yogurt: received yogurt with *B. coagulans* GBI-30 6086.

At the order level, the majority of OTU in the six treatments (Yogurt, Probiotic Yogurt, Juice, Probiotic Juice, Probiotic *Bacillus*, and Control) were attributed to three significant orders: *Clostridiales* (from 46.3 to 51.8%), *Bacteroidales* (from 33.7 to 43.3%), and *Lactobacillales* (from 5.1 to 6.8%; Figure 2; Table 5). When the abundance was observed without these three major groups (i.e., *Clostridiales, Bacteroidales*, and *Lactobacillales*), most OTUs were attributed to the same orders in all groups. However, Probiotic Yogurt samples showed a higher (*p* < 0.05) abundance of *Vibrionales* (2.2%), *Enterobacteriales* (2.1%), *Burkholderiales* (1.6%), *Erysipelotrichales* (0.5%), and *Bifidobacteriales* (0.1%) when compared to the other groups (Figure 2; Table 5).

**Table 5:** Relative abundance of bacterial orders inferred from 16S rRNA gene sequencing analysis.^*^

Taxonomy (%)	Control	Juice	Yogurt	Probiotic *Bacillus*	Probiotic juice	Probiotic yogurt
*Clostridiales*	47.4 ± 4.7^a^	49.1 ± 2.1^a^	51.8 ± 2.5^a^	47.3 ± 4.3^a^	46.8 ± 3.2^a^	46.3 ± 3.7^a^
*Bacteroidales*	39.0 ± 3.9^a^	38.9 ± 2.1^a^	33.7 ± 2.5^a^	41.5 ± 3.6^a^	43.3 ± 3.1^a^	39.3 ± 2.3^a^
*Lactobacillales*	7.4 ± 1.8^a^	7.7 ± 1.2^a^	8.9 ± 0.65^a^	7.1 ± 1.07^a^	6.8 ± 0.52^a^	5.1 ± 1.1^a^
*Bacillales*	0.08 ± 0.02^a^	0.1 ± 0.00^ab^	0.14 ± 0.02^ab^	0.14 ± 0.02^ab^	0.14 ± 0.02^ab^	0.16 ± 0.009^b^
*Bifidobacteriales*	0.02 ± 0.02^a^	0.00 ± 0.00^a^	0.00 ± 0.00^a^	0.00 ± 0.00^a^	0.00 ± 0.00^a^	0.10 ± 0.05^b^
*Coriobacteriales*	0.10 ± 0.00^a^	0.12 ± 0.03^a^	0.10 ± 0.03^a^	0.10 ± 0.00^a^	0.08 ± 0.02^a^	0.14 ± 0.02^a^
*Verrucomicrobiales*	0.00 ± 0.00^a^	0.00 ± 0.00^a^	0.96 ± 0.96^a^	0.94 ± 0.91^a^	0.02 ± 0.02^a^	0.04 ± 0.02^a^
*Mollicutes*	0.34 ± 0.15^a^	0.32 ± 0.10^a^	0.86 ± 0.54^a^	0.22 ± 0.06^a^	0.30 ± 0.10^a^	0.28 ± 0.10^a^
*Unassigned;Other*	0.38 ± 0.06^a^	0.42 ± 0.02^a^	0.42 ± 0.03^a^	0.44 ± 0.04^a^	0.48 ± 0.03^a^	0.40 ± 0.03^a^
*Cyanobacteria*	0.56 ± 0.17^a^	0.57 ± 0.38^a^	0.42 ± 0.25^a^	0.22 ± 0.07^a^	0.26 ± 0.08^a^	0.14 ± 0.06^a^
*Burkholderiales*	0.42 ± 0.09^a^	0.65 ± 0.19^a^	0.32 ± 0.09^a^	0.34 ± 0.09^a^	0.24 ± 0.02^a^	1.62 ± 0.55^a^
*Desulfovibrionales*	0.18 ± 0.03^a^	0.27 ± 0.06^a^	0.26 ± 0.07^a^	0.34 ± 0.07^a^	0.24 ± 0.05^a^	0.22 ± 0.03^a^
*Campylobacterales*	0.34 ± 0.11^a^	0.25 ± 0.09^a^	0.16 ± 0.06^a^	0.38 ± 0.19^a^	0.40 ± 0.18^a^	0.20 ± 0.03^a^
*Enterobacteriales*	0.20 ± 0.00^a^	0.25 ± 0.08^a^	0.20 ± 0.03^a^	0.20 ± 0.07^a^	0.14 ± 0.02^a^	2.1 ± 0.89^b^
*Turicibacterales*	0.20 ± 0.09^ab^	0.10 ± 0.04^ab^	0.06 ± 0.04^ab^	0.02 ± 0.02^a^	0.08 ± 0.02^ab^	0.22 ± 0.07^b^
*Erysipelotrichales*	0.18 ± 0.04^a^	0.47 ± 0.13^b^	0.10 ± 0.00^a^	0.12 ± 0.04^a^	0.12 ± 0.04^a^	0.30 ± 0.15^ab^
*Vibrionales*	0.10 ± 0.00^a^	0.10 ± 0.00^a^	0.08 ± 0.02^a^	0.08 ± 0.03^a^	0.06 ± 0.02^a^	2.1 ± 1.0^b^
*Alteromonadales*	0.00 ± 0.00^a^	0.00 ± 0.00^a^	0.00 ± 0.00^a^	0.00 ± 0.00^a^	0.00 ± 0.00^a^	0.06 ± 0.02^b^

* Groups were as follows: Control: received distilled water; Juice: received orange juice; Yogurt: received yogurt; probiotic *Bacillus*: received *B. coagulans* GBI-30 6086 suspended in distilled water; Probiotic Juice: received orange juice with *B. coagulans* GBI-30 6086; and Probiotic Yogurt: received yogurt with *B. coagulans* GBI-30 6086; Data are expressed as means ± SEM. Different superscript letters on the same line indicate statistical differences by the Duncan test (*p* < 0.05).

**Figure 3:**
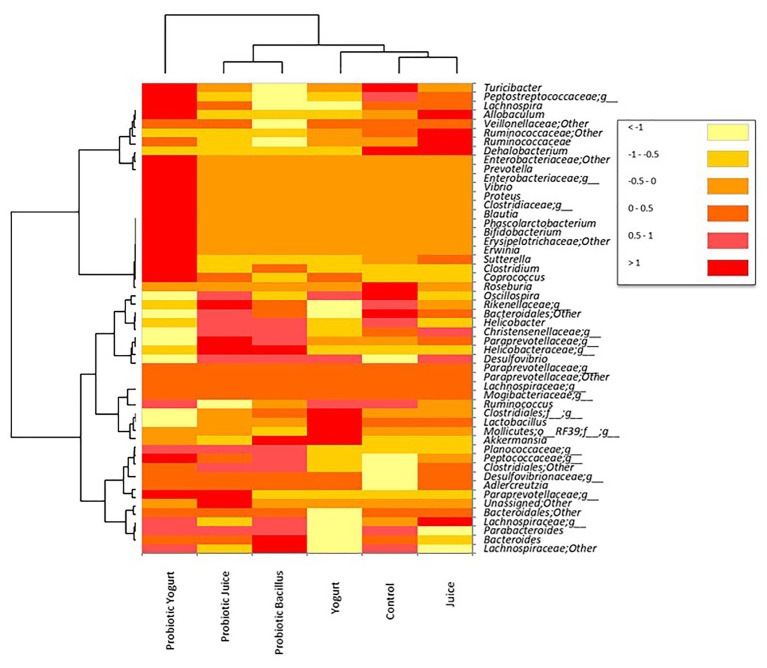
Heatmap showing microbial taxa (mostly family level) with relative abundance obtained by 16S rRNA gene pyrosequencing analysis. Groups were as follows: Control: received distilled water; Juice: received orange juice; Yogurt: received yogurt; probiotic *Bacillus*: received *B. coagulans* GBI-30 6086 suspended in distilled water; Probiotic Juice: received orange juice with *B. coagulans* GBI-30 6086; and Probiotic Yogurt: received yogurt with *B. coagulans* GBI-30 6086. Only OTUs with abundance values above 0.1% in at least nine readings are shown.

A heat map analysis was performed to explore the taxonomic assignment obtained by 16S rRNA gene sequencing analysis and better visualize the similarities and differences in each bacterial affiliation among the treatments (Figure 3). As can be seen, the heat map presented two more prominent clusters, one composed of the Probiotic Juice and Probiotic *Bacillus* samples and the other by the Control and Juice samples. Besides, it was observed that the Probiotic Yogurt samples clustered completely separated (Figure 3). The microbial diversity of the samples was significantly different between each treatment. However, it was observed that the Probiotic juice and Probiotic *Bacillus* samples presented similar patterns concerning specific microbial groups: *Helicobacter, Christensenellaceae;g_, Paraprevotellaceae;g_, Helicobacteraceae;g_; Planococcaceae;g_, Clostridiales*; Other (Figure 2). On the other hand, the Probiotic Yogurt samples showed a high abundance of specific microbial groups than other treatments, such as *Turicibacter, Peptostreptococcaceae:g_, Lachnospira, Allobaculum, Enterobacteriaceae; Other, Prevotella; Enterobacteriaceae;g_, Vibrio, Proteus, Clostridiaceae;g_, Blautia, Phascolarctobacterium, Bifidobacterium, Erysipelotrichaceae*; Other, *Erwinia, Sutterella, Clostridium, Coprococcus, and Peptococcaceae*;g_. The Juice and Control samples presented the abundance of the *Dehalobacterium* genus.

## Discussion

Despite the no surprising lack of chemical variations due to *Bacillus coagulans* GBI-30, 6086 in the studied matrices since the spores are metabolically inactive cells, these results are essential to clarify that the effects observed were not related to chemical changes in the matrix caused by the probiotic.

In recent years, a range of studies in animal models has reported beneficial effects of probiotics (added or not to food matrices) to the host’s health, such as intestinal microbiota modulation (Marchesin et al., 2018), alleviation of inflammation (Mousavi et al., 2020) and food allergy (Maa et al., 2019), improvement of the immune system (Manuel et al., 2017), relief of symptoms caused by cardiovascular disorders (Cavalcante et al., 2019), diabetes Type 2 (Wang et al., 2020a), and colorectal cancer (Genaro et al., 2019), among others. However, few studies have focused on evaluating spore-forming probiotics effects through food consumption (Sun et al., 2011; Ng et al., 2013; Haldar and Gandhi, 2016). Therefore, the current study evaluated the effects of the probiotic spore-forming *B. coagulans* GBI-30 6086 on biochemical parameters and gut microbiota profile in healthy rats. This study also revealed the effects of the food matrices on the probiotic spore-forming *B. coagulans* GBI-30 6086 functionality.

Our results showed that the administration of the tested strain in water, juice, or yogurt for 21 days does not affect the food intake or weight gain in animals. Overall, daily ingestion of probiotic yogurt decreased serum triglycerides and glucose, while these same effects were not observed for the probiotic juice’s daily ingestion. It is important to point out that the differences in the yogurt and orange juice’s chemical composition can affect these biochemical parameters. Orange juice showed a higher carbohydrate content, while yogurt has a higher content of fat and proteins. The type of sugars present in each food matrix should also be considered. Orange juice stands out for its greater fructose presence, while yogurt for glucose and galactose (Ranadheera et al., 2010).

Increased triglycerides and glucose levels are risk factors associated with the development of coronary heart disease and diabetes mellitus, respectively (Karamali et al., 2016; Wang et al., 2020a). Therefore, there is a growing interest in probiotic foods that did not affect the food intake besides exerting positive effects of lipids and glucose metabolism, as observed for the probiotic yogurt in the present study. Lipids and glucose blood levels are overall classical biochemical markers elevated in animals with metabolic disorders (Roquetto et al., 2015; Costa et al., 2019). It is believed that if probiotic yogurt consumption reduced these parameters in healthy animals, they could also be attenuated when increased in the blood.

In contrast to lowering-cholesterol effects observed for probiotic yogurt (containing *L. acidophilus* e *Bifidobacterium lactis*) in hypercholesterolemic subjects (Ataie-Jafari et al., 2009), no prior studies reported effects of yogurt with PB on the serum lipid profile. In the meanwhile, the administration of non-sporulated probiotic strains (*Lactilactobacillus curvatus* HY7601 and *Lactiplantibacillus plantarum* KY1032) led to a reduction of 18% in the serum triglycerides in non-diabetic subjects with mild to moderate hypertriglyceridemia (Ahn et al., 2015), similar to the observed effects in our study by the administration of the probiotic *Bacillus* suspension.

The improvement of the glycemic and lipid parameters by probiotic strains has been primarily associated with the restoration of the gut barrier function through colonization (Wang et al., 2020a). The ability of non-spore-forming probiotic [*Lactobacillus* and amended genera strains (*Lactiplantibacillus plantarum*, *Lactobacillus helveticus*, *Lactiplantibacillus pentosus*, *Lacticaseibacillus casei*, *Limosilactobacillus mucosae*, *Lacticaseibacillus rhamnosus*, *Schleiferilactobacillus harbinensis*, and *Lentilactobacillus hilgardii*) and yeasts (*Issatchenkia orientalis*, *Candida ethanolica*, *Kluyveromyces marxianus*, and *Pichia membranifaciens*)] to reduce the blood glucose in diabetic mice have been suggested through directly glucose metabolism in the gut (Wang et al., 2020b). Inhibition of the *α*-glucosidase, which hydrolyzes glycosidic bonds releasing glucose, is also considered a *probiotic* (*Lacticaseibacillus rhamnosus, Lactobacillus acidophilus, Lactiplantibacillus plantarum, Bifidobacterium animalis, Bifidobacterium longun*) to regulate glucose metabolism in the blood of diabetic mice (Wang et al., 2020a). Other reports account the beneficial effects of probiotics (*Bifidobacterium, Lactobacillus*, and amended genera) on the control of glycemia and triglycerides levels to increase in hepatic natural-killer-cells, reduction of inflammatory signaling (Ma et al., 2008), up-regulation of adiponectin (Nakamura and Omaye, 2012), and increase glucagon-like peptide (GLP)-1that influence the improvement of carbohydrate metabolism (Tremaroli and Bäckhed, 2012). However, the studies have assessed these features in non-sporulated probiotics. Thus, the underlying mechanism of PB remains unclear. It can be suggested that similar mechanisms are involved in PB effects. However, experimental studies are needed to prove this relationship.

Probiotics may exert antioxidant activity through enzyme activation to protect cells against oxidative stress (Petrof et al., 2004), while heat shock proteins (HSPs) play critical roles in the regulation of both acute and chronic stresses (Zininga et al., 2018). Particularly, HSP-70 has a cytoprotective action against structural and functional damage induced by oxidative stress and inflammation. Both antioxidant enzymes and HSP help maintain homeostasis, vital for the intestinal barrier function (Arnal and Lallès, 2014). Consumption of *Bacillus coagulans* GBI-30 6086 either in distilled water or incorporated in food matrices (yogurt or juice) did not affect antioxidant enzymes and HSP expression to observed by Moura et al. (2016) in an experiment with *Lactobacillus acidophilus* LA 05 incorporated in a dairy dessert. The possible relationship between probiotics and HSPs is not yet elucidated, but it seems that low molecular weight peptides and other soluble factors secreted by probiotics in the intestinal lumen would modulate the expression of HSPs (Tao, 2005). Since HSPs are usually expressed in stress situations and act as a cellular defense, a healthy model may be related to the lack of changes in these parameters. Additionally, a limited number of probiotic strains may reduce oxidative stress (Kleniewska et al., 2016). These results are significant because they show that the amount of ingested probiotic or probiotic food did not increase the animals’ stress.

Analyses of gut microbiota showed that the probiotic yogurt consumption resulted in a higher abundance and diversity of male rats’ gut microbiota profile than the other samples. Lactic acid bacteria’s presence, which is used as starter cultures for yogurt production, may influence these findings because they may have a synergistic effect with the probiotic *B. coagulans* modifying the microbiome composition (Ranadheera et al., 2010). Otherwise, *Clostridiales, Bacteroidales*, and *Lactobacillales* were not significantly altered among the six groups studied. Alteration in these microbial groups has been associated with chronic diseases (Ma et al., 2020). Thus, these are results that can be considered favorable for both the Probiotic juice and Probiotic Yogurt.

The ingestion of the probiotic yogurt caused an increase of specific orders such as *Bifidobacteriales* and *Bacillales*, which were not increased by the ingestion of the probiotic juice, showing a positive gut microbiota modulation by probiotic yogurt, the influence of the food carrier on these effects.

*Bifidobacteriales* are considered one of the main groups, including bacterial members, exhibiting probiotic health-promoting effects in humans (Zhang et al., 2016), while several groups in *Bacillales* also have remarkable health-beneficial properties (Cao et al., 2020). The increase observed in *Bacillales* by the ingestion of yogurt with *B. coagulans* GBI-30 6086 suggests that this matrix delivered the strain in the gut, where the PB germinated, grew, and multiplied as a vegetative form enabling the adhesion to the intestine and exert beneficial effects (Ghelardi et al., 2015; Haldar and Gandhi, 2016). A previous study reported that the spore germination in *Bacillus* strains with further metabolic activity in the gut is influenced by the environmental conditions (Bernardeau et al., 2017) and, as observed here, by the food matrix carrying the spores.

The gut microbiota profile changes in this study are consistent with the results of another study with *Bacillus* strains (*B. coagulans* B37 and *B. pumilus* B9) in skim milk increased lactobacilli and *Bacillus* spp. in the intestinal microbiota in rats (Haldar and Gandhi, 2016). Otherwise, Chaikham et al. (2012) observed that juice added with non-sporulated probiotics (*Lactobacillus acidophilus* LA5 or *Lacticaseibacillus casei* 01) modulated the intestinal microbiota, increasing *Bifidobacteria* and decreasing pathogenic bacteria (e.g., *Clostridia* and fecal coliforms). However, the study was performed *in vitro*, precluding a direct comparison since the environment exerts potent effects on probiotic effects.

Even though *B. coagulans* GBI-30 6086 was added to food matrices as spores, the results of this study clearly show that the food matrix is also relevant for delivering the spore-forming probiotic bacteria. Yogurt was a better carrier of *B. coagulans* GBI-30 6086 compared to orange juice, which is likely due to the yogurt’s chemical composition and the presence of lactic bacteria. Yogurt has a higher fat and protein content than juice, while juice content in carbohydrates is higher (Supplementary Table 1). Probably these characteristics interfere with the efficacy of the matrix as carrier and maintenance of vegetative cells in the intestine through the interaction of these components with the probiotic cells, boosting its beneficial effects (Ranadheera et al., 2010).

## Conclusion

Results obtained in the present study show that the daily consumption of yogurt containing *B. coagulans* GBI-30 6086 during 21 days decreases the glucose and triglycerides serum levels in healthy rats and positively modulated the gut microbiota by increasing *Bacillales* and *Bifidobacteriales*. These findings indicate yogurt as an efficient food carrier to deliver probiotic spore-forming bacteria and suggest that yogurt consumption containing *B. coagulans* GBI-30 6086 can be an important dietary strategy to reduce biochemical markers associated with metabolic diseases and modulate the gut microbiota ecology. These animal models’ findings indicate that the food matrix impacts spore-forming probiotics’ functionality and suggests yogurt as a suitable food carrier of probiotic *Bacillus*.

## Data Availability Statement

The datasets presented in this study can be found in online repositories. The names of the repository/repositories and accession number(s) can be found at: https://www.ncbi.nlm.nih.gov/, PRJNA631217.

## Ethics Statement

The animal study was reviewed and approved by Ethical Commission on Animal Use (CEUA, UNICAMP, São Paulo, Brazil, protocol n° 3456-1).

## Author Contributions

ASS and CA-E conceived the study. ASS, CA-E, CA, AR, and PL designed the experiments. CA-E, CA, AR, VS-J, and AG conducted the experiments. CA-E, CA, AR, VS-J, LC, MF, AG, AS, PL, VB, and MM analyzed the results. CA-E, CA, LC, VB, MM, and ASS drafted the manuscript. CA-E, CA, LC, AG, PL, AS, VB, MM, and ASS revised the manuscript. All authors contributed to the article and approved the submitted version.

### Conflict of Interest

The authors declare that the research was conducted in the absence of any commercial or financial relationships that could be construed as a potential conflict of interest.

## Footnotes

^1^ncbi.nlm.nih.gov
