# Supplementary material for: Orange Juice and Yogurt Carrying Probiotic Bacillus coagulans GBI-30 6086: Impact of Intake on Wistar Male Rats Health Parameters and Gut Bacterial Diversity
Source: Front Microbiol. 2021 Apr 1;12:623951. doi: 10.3389/fmicb.2021.623951 (PMC8202523; doi:10.3389/fmicb.2021.623951)
Supplement: Supplementary file 1 [file Table_1.docx]

**TABLE S1**. Chemical composition of juice, probiotic juice, yogurt and probiotic yogurt.

| **Component**  **(g/100 mL)** | **Juice** | **Probiotic juice** | **Yogurt** | **Probiotic yogurt** |
| --- | --- | --- | --- | --- |
| Moisture | 91.5±0.95 ^aA^ | 91.9±0.04 ^aA^ | 87.9±0.09 ^aA^ | 87.9±0.20^aA^ |
| Ashes | 0.43±0.01 ^aA^ | 0.60±0.00 ^aA^ | 0.82±0.01 ^aB^ | 0.88±0.02 ^aB^ |
| Total lipids | ND (˂0.10) ^bA^ | ND (˂0.10) ^bA^ | 2.8±0.01 ^aB^ | 2.8±0.03 ^aB^ |
| Proteins | 0.79±0.00 ^aA^ | 0.82±0.00 ^aA^ | 3.3±0.01 ^aB^ | 3.4±0.02 ^aB^ |
| Total carbohydrates* | 11.2^A^ | 11.6^A^ | 5.0^B^ | 4.8^B^ |

^a^ Data are expressed as means ± SD. ^b^ ND= Not detected; * total carbohydrates inferred by difference. Different superscript capital letters on the same line indicate statistical differences by Scott-Knott test (p ˂ 0.05).
