# Supplementary material for: Orange Juice and Yogurt Carrying Probiotic Bacillus coagulans GBI-30 6086: Impact of Intake on Wistar Male Rats Health Parameters and Gut Bacterial Diversity
Source: Front Microbiol. 2021 Apr 1;12:623951. doi: 10.3389/fmicb.2021.623951 (PMC8202523; doi:10.3389/fmicb.2021.623951)
Supplement: Supplementary file 2 [file Table_2.docx]

| **Treatment code*** | **NCBI Biosample acession** | **Sequence** | **Quality-filtering** |
| --- | --- | --- | --- |
| Control | SAMN14830232 | 480592 | 451812 |
|  | SAMN14830300 | 815752 | 751936 |
|  | SAMN14830392 | 256840 | 239876 |
|  | SAMN14830574 | 895960 | 845316 |
|  | SAMN14830654 | 875656 | 826860 |
| Juice | SAMN14830705 | 540120 | 508728 |
|  | SAMN14830780 | 821560 | 755200 |
|  | SAMN14830821 | 578912 | 534908 |
|  | SAMN14830886 | 943432 | 889184 |
| Yogurt | SAMN14831058 | 1318448 | 1221844 |
|  | SAMN14831230 | 654360 | 613972 |
|  | SAMN14831233 | 584016 | 548396 |
|  | SAMN14831248 | 427272 | 401964 |
|  | SAMN14831284 | 1142416 | 1051588 |
| Probiotic *Bacillus* | SAMN14832254 | 211768 | 197644 |
|  | SAMN14832255 | 599712 | 564116 |
|  | SAMN14832256 | 338128 | 316756 |
|  | SAMN14832257 | 753768 | 709700 |
|  | SAMN14832262 | 531016 | 482368 |
| Probiotic Juice | SAMN14844537 | 467520 | 430536 |
|  | SAMN14844538 | 909296 | 852076 |
|  | SAMN14844540 | 281248 | 256632 |
|  | SAMN14844541 | 427552 | 399992 |
|  | SAMN14844551 | 687592 | 648380 |
| Probiotic Yogurt | SAMN14844554 | 1782624 | 1658656 |
|  | SAMN14844567 | 1831744 | 1501092 |
|  | SAMN14844568 | 958440 | 884372 |
|  | SAMN14844569 | 703376 | 637452 |
|  | SAMN14844570 | 646912 | 590132 |
| TOTAL OF SEQUENCES | | 21466032 | 19771488 |

**TABLE S2.** Samples, NCBI Biosample acession and sequences quality filters applied by Trimmomatic (0.36) software.

*Groups were as follow: Control: received distilled water; Juice: received orange juice; Yogurt: received yogurt; probiotic *Bacillus*: received *B. coagulans* GBI-30 6086 suspended in distilled water; Probiotic juice: received orange juice with *B. coagulans* GBI-30 6086 and, Probiotic yogurt: received yogurt with *B. coagulans* GBI-30 6086.
