# Supplementary material for: Orange Juice and Yogurt Carrying Probiotic Bacillus coagulans GBI-30 6086: Impact of Intake on Wistar Male Rats Health Parameters and Gut Bacterial Diversity
Source: Front Microbiol. 2021 Apr 1;12:623951. doi: 10.3389/fmicb.2021.623951 (PMC8202523; doi:10.3389/fmicb.2021.623951)
Supplement: Supplementary file 3 [file Table_3.docx]

**Table S3.** Kruskal – Wallis test using OTUs and alpha diversity indexes (Chao1, Shannon and Simpson) obtained for all samples.

| **Comparison** | **Chi-squared** | **df** | ***p-value*** |
| --- | --- | --- | --- |
| OTUs | 12.462 | 5 | 0.02897* |
| Chao1 | 13.448 | 5 | 0.01952* |
| Shannon | 12.188 | 5 | 0.0323* |
| Simpson | 6.844 | 5 | 0.2325 |

* *p-values* reported in Table 5 were calculated using the Kruskal – Wallis test (*p* < 0.05).
