# Supplementary material for: Orange Juice and Yogurt Carrying Probiotic Bacillus coagulans GBI-30 6086: Impact of Intake on Wistar Male Rats Health Parameters and Gut Bacterial Diversity
Source: Front Microbiol. 2021 Apr 1;12:623951. doi: 10.3389/fmicb.2021.623951 (PMC8202523; doi:10.3389/fmicb.2021.623951)
Supplement: Supplementary file 4 [file Table_4.docx]

**Table S4.** Permanova statistical analysis from beta-diversity data using Adonis script in QIIME.

| *opts$category* | Df | SumsOfSqs | MeanSqs | F.Model | R2 Pr(>F) | Pr(>F) |
| --- | --- | --- | --- | --- | --- | --- |
| qiime.data$map | 5 | 1.2246 | 0.24491 | 1.0622 | 0.18759 | 0.002 ** |
| Residuals | 23 | 5.3034 | 0.23058 |  | 0.81241 |  |
| Total | 28 | 6.5280 |  |  | 1.00000 |  |

Signif. codes: 0 ‘***’ 0.001 ‘**’ 0.01 ‘*’ 0.05 ‘.’ 0.1 ‘ ’ 1
