# Supplementary material for: Orange Juice and Yogurt Carrying Probiotic Bacillus coagulans GBI-30 6086: Impact of Intake on Wistar Male Rats Health Parameters and Gut Bacterial Diversity
Source: Front Microbiol. 2021 Apr 1;12:623951. doi: 10.3389/fmicb.2021.623951 (PMC8202523; doi:10.3389/fmicb.2021.623951)
Supplement: Supplementary file 5 [file Data_Sheet_1.docx]

Fig. S1 Effect of administration of probiotic yogurt and probiotic juice during 21 days on expression of antioxidant enzymes and heat shock protein (HSP) 70 in healthy *Wistar* male rats relative to control assessed in Western Blot assays.


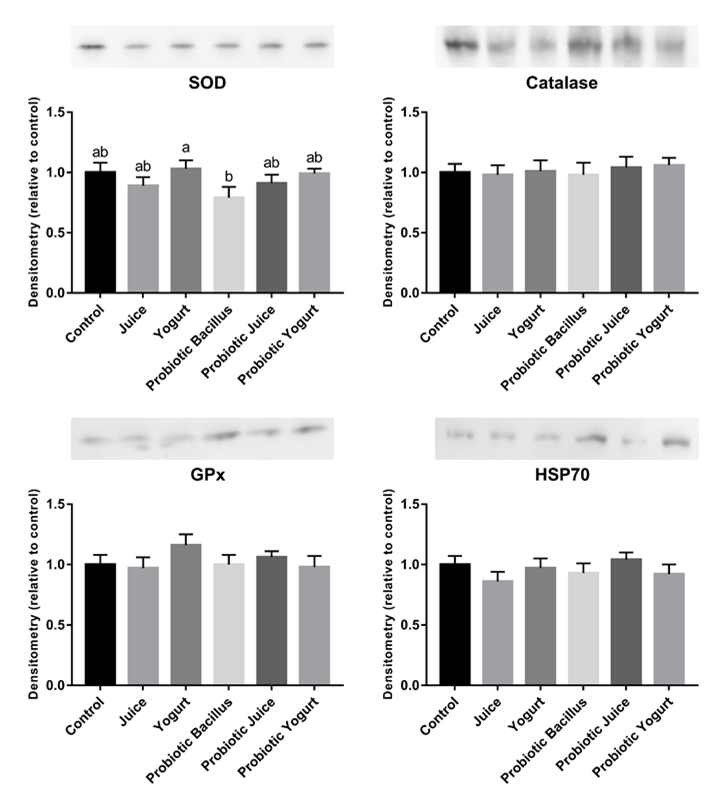


*Groups were as follow: Control: received distilled water; Juice: received orange juice; Yogurt: received yogurt; probiotic *Bacillus*: received *B. coagulans* GBI-30 6086 suspended in distilled water; Probiotic juice: received orange juice with *B. coagulans* GBI-30 6086 and, Probiotic yogurt: received yogurt with *B. coagulans* GBI-30 6086. Data are expressed as means ± SEM. Different letters indicate statistical differences by Duncan test (p ˂ 0.05).
